# CricRNA in head and neck squamous cell carcinoma: biological functions and clinical prospects

**DOI:** 10.3389/fonc.2025.1692671

**Published:** 2025-11-05

**Authors:** Anqi Li, Zhen Dong, Chunming Zhang

**Affiliations:** Department of Otorhinolaryngology-Head and Neck Surgery, The First Hospital of Shanxi Medical University, Taiyuan, Shanxi, China

**Keywords:** circular RNA, head and neck squamous cell carcinoma, biomarker, tumor progression, non-coding RNA, therapeutic target, translational medicine

## Abstract

Head and neck squamous cell carcinoma (HNSCC) is a prevalent malignant tumor globally, shows an unfavorable prognosis and has a five - year survival rate lower than 50%. Circular RNA (circRNA) is defined as a type of non-coding RNA molecule that forms a covalently closed circular structure through back-splicing, characterized by tissue specificity, high stability, and stability in body fluid detection. Recent research has frequently revealed marked alterations in circRNA regulation in HNSCC and participates in tumor malignant behaviors such as cell proliferation and apoptosis, invasion and metastasis, epithelial-mesenchymal transition (EMT), chemoresistance, and immune evasion through mechanisms like “miRNA sponges,” protein scaffolding, translation templates, and epigenetic regulation. This review aims to provide a systematic and up-to-date consolidation of the latest advances in circRNA research for HNSCC, from its biogenesis and multifaceted functions to clinical translation. Our goal is to uncover novel biomarkers and therapeutic targets for the precise diagnosis and management of HNSCC. This review not only aids in elucidating the complex regulatory networks orchestrated by circRNAs in HNSCC but also highlights their immense potential as novel diagnostic and therapeutic agents, thereby paving the way for future research and clinical application.

## 1 Overview of circRNA

### 1.1 Biogenesis and classification

**Figure 1:**
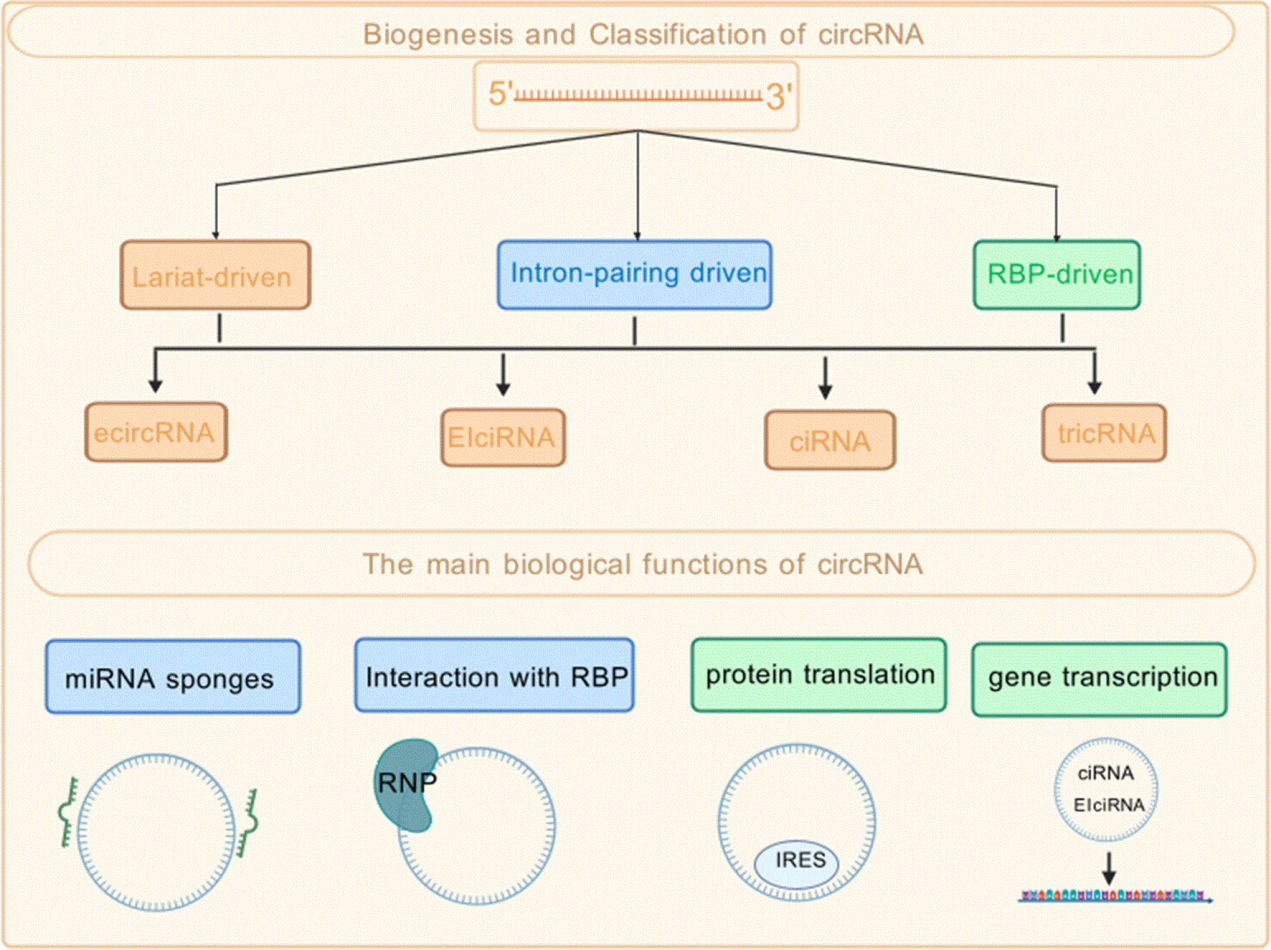
Biogenesis, classification, and functional mechanisms of circRNAs.

Sanger et al. first detected circular RNA (circRNA) within viroids in 1976. RNA was then classified as non-translational RNA (1). These molecules are characterized by widespread distribution, strong structure, and remarkable conservatism (2). In the human body, Circular RNAs are detected in almost all tissues. However, different circRNAs have expression profiles that are specific to distinct tissues or different developmental stages (3). Circular RNAs (circRNAs) originate from precursor mRNA (pre-mRNA) or lncRNA through a process known as back-splicing. According to the differences in their splicing mechanisms, The primary classification of circular RNAs (circRNAs) comprises three categories. The first category is exon circular RNA, which is commonly abbreviated as ecircRNA; the second type is exon-intron circular RNA, with its typical abbreviation being EIciRNA; and the third category is intron circular RNA, usually referred to as ciRNA for short. Each of these three categories exhibits distinct structural characteristics that are closely associated with their respective splicing processes, thereby forming the main classification system of circRNAs based on splicing mechanism differences (4). **Figure 1** summarizes the biogenesis pathways and major functional mechanisms of circRNAs.

### 1.2 Biological functions

#### 1.2.1 miRNA sponge

Research has demonstrated that a single circRNA can function as a sponge for miRNAs. Under certain circumstances, it can act not only as a tumor suppressor but also as an oncogene. As a case in point, circHIPK3 exhibits the capability to function as a molecular sponge. Its target is miR-558, a type of microRNA. Through this sponging effect, circHIPK3 exerts an inhibitory impact (5).Specifically, it suppresses the expression level of heparanase. This regulatory pathway takes place in the context of bladder cancer. Consequently, this elevation of STAT3 expression further facilitates the initiation and development of lung cancer (6).

#### 1.2.2 Protein scaffold

circRNA can also function as a protein scaffold, promoting chemical reactions or inhibiting protein functions. For instance, in mouse fibroblasts, circ-Foxo3 is able to interact with p21 (a cyclin-dependent kinase [CDK] inhibitor) and cyclin-dependent kinase 2 (Cdk2), forming a three-component complex that suppresses cell cycle progression (7). Furthermore, circ-Foxo3 can serve as a scaffold for the mouse proteins Mdm2 and p53, in turn facilitating the degradation of p53 (8).

#### 1.2.3 Translation template

Research has demonstrated that numerous circRNAs contain internal ribosome entry sites (IRES) or open reading frames (ORFs). Through these elements, these circRNAs are involved in the transcription and translation processes of functional proteins. Zhang et al. has been reported that circ-SHPRH encodes the protein SHPRH-146aa, which acts as a decoy, protecting the SHPRH protein from ubiquitin-mediated degradation by retinoic acid-regulated nuclear matrix-associated protein (DTL), thereby inhibiting glioma development (9).

#### 1.2.4 Epigenetic regulation

Zhu et al. found a close association between CircSEPT9 and miR-10a in LSCC samples. In LSCC cells, the overexpression of circSEPT9 enhances the methylation level of the miR-10a gene. This increase in miR-10a gene methylation, in turn, leads resulting in a reduction in the expression of miR-10a.Both overexpression of circSEPT9 and miR-10a increased cell proliferation in LSCC. Co-transfection experiments suggested that the overexpression of circSEPT9 diminished the effect of miR-10a overexpression. The study concluded that circSEPT9 may increase miR-10a methylation, thereby promoting cell proliferation in LSCC (10).

### 1.3 Stability and distribution advantages

The covalently closed loop confers resistance to exonucleases, and several studies have reported prolonged half-lives for select circRNAs compared with their linear host transcripts in cell-culture and plasma settings; systematic comparative data across all circRNA species are still lacking. CircRNA expression is highly conservative and spatially and temporally specific, with its type and abundance varying significantly across different tissues, cells, and developmental stages (11),CircRNA exhibits distinct expression patterns that are tissue-specific Besides, it also exhibits distinct expression patterns related to developmental stages. Moreover, it (circRNA) can be detected stably in a range of body fluids. Examples of such fluids are plasma, serum, saliva, cerebrospinal fluid, and gastric juice. It mainly occurs in the form of circular RNA encapsulated in exosomes (exo-circRNA) or cell-free circular RNA (cf-circRNA), which creates the conditions for non-invasive diagnosis (12).

## 2 Expression profiles of circRNA in HNSCC

### 2.1 High-throughput sequencing results

Wang et al. (2018) conducted high-throughput microarray analysis to detect circRNA and mRNA expression in HNSCC tissues. In this study, microarray probes detected a total of 12,366 circRNAs and 35,252 mRNAs in 5 pairs of HNSCC and normal tissues. Using a discovery microarray (5 tumor-normal pairs, FDR < 0.05), they detected 287 circRNAs and 1,053 mRNAs as differentially expressed candidates; independent, larger cohorts are required to confirm these findings. Of these mRNAs, 377 showed upregulated expression and 676 displayed downregulated expression (13).

As an example, Wu et al. identified 139,643 human and 214,747 mouse circRNAs in single-cell RNA sequencing (scRNA-seq) libraries. They then integrated 11 RNA-based bulk RNA-seq resources to validate these detected circRNAs. Through this validation, the single-cell cohorts allowed for the unique detection of 216,602 high-confidence circRNAs. The research identified cell type-specific expression profiles of circRNAs across three distinct biological contexts (developing embryos, brain specimens, and breast tumor specimens) while also identifying circRNAs with unique expression profiles in different cell types, and it further validated the functional relevance of these uniquely expressed circRNAs—specifically their utility in the deconvolution of immune cells infiltrating tumors. By pushing the analysis of circRNA expression at the single-cell resolution, this study provides a valuable resource that supports the further investigation of circRNAs (14).

### 2.2 Database resources

MNDR v3.0 (Mammal ncRNA-Disease Repository version 3.0) is a database specifically dedicated to collecting and organizing the relationships between mammalian non-coding RNAs (ncRNAs) and diseases. This database combines both experimentally confirmed and predicted associations between circular RNAs (circRNAs) and diseases, expanding the range of mammalian species covered in the study to 11. This resource encompasses 6,301 non-redundant microRNAs (miRNAs), 39,880 long non-coding RNAs (lncRNAs), 20,506 circular RNAs (circRNAs), 10,894 Piwi-interacting RNAs (piRNAs), and 521 small nucleolar RNAs (snoRNAs), while the number of covered disease types has been elevated to 1,614. In addition, this resource offers comments on relevant drugs and incorporates four categories of non-coding RNA (ncRNA)-drug relationships, namely drug targets, drug sensitivity, drug resistance, and drug interactions (15).

MNDR v3.0 further offers three ncRNA-disease prediction tools via its official website, with one of them being the structural perturbation method (SPM)—a tool specifically designed for miRNA-disease prediction, sparse inductive matrix completion with latent Dirichlet allocations (SIMCLDA) for predicting lncRNA diseases, and deep forest with positive unlabeled learning (DeepDCR) for calculating the relationships between circRNAs and diseases (16). Utilizing the RNADisease website (http://www.rnadisease.org/prediction), we conducted a disease association analysis for specific circRNAs. The results indicated that hsa_circ_0000237 exhibited a score of 7.5200E-5 for oral squamous cell carcinoma and 1.6800E-5 for laryngeal squamous cell carcinoma. Additionally, hsa_circ_0000190 obtained a score of 8.7200E-5 for oral squamous cell carcinoma. It is crucial to note that these data are derived from computational model predictions and may be supported by limited experimental evidence. Therefore, further experimental validation is imperative to substantiate these associations and elucidate the underlying mechanisms.

## 3 Molecular mechanisms of circRNA-driven malignant phenotypes in HNSCC

### 3.1 Cell proliferation and cycle

Verduci L (2017) collected samples from 115 patients with HNSCC and observed that circPVT1 exhibited significantly higher expression in tumor tissues than in their matched tumor-free counterparts, with this overexpression being particularly prominent in patients carrying TP53 mutations. The upregulation or downregulation of circPVT1 in HNSCC cell lines was found to be associated with a corresponding elevation or reduction in the malignant phenotype of these cells. As documented in the study, the mut-p53/YAP/TEAD complex was responsible for mediating an increase in circPVT1 expression (17). Observational and functional data suggest that circPVT1 may exert oncogenic activity in part through relief of miR-497-5p-mediated growth inhibition; prospective and loss-of-function studies *in vivo* are warranted to establish causality.

**Figure 2:**
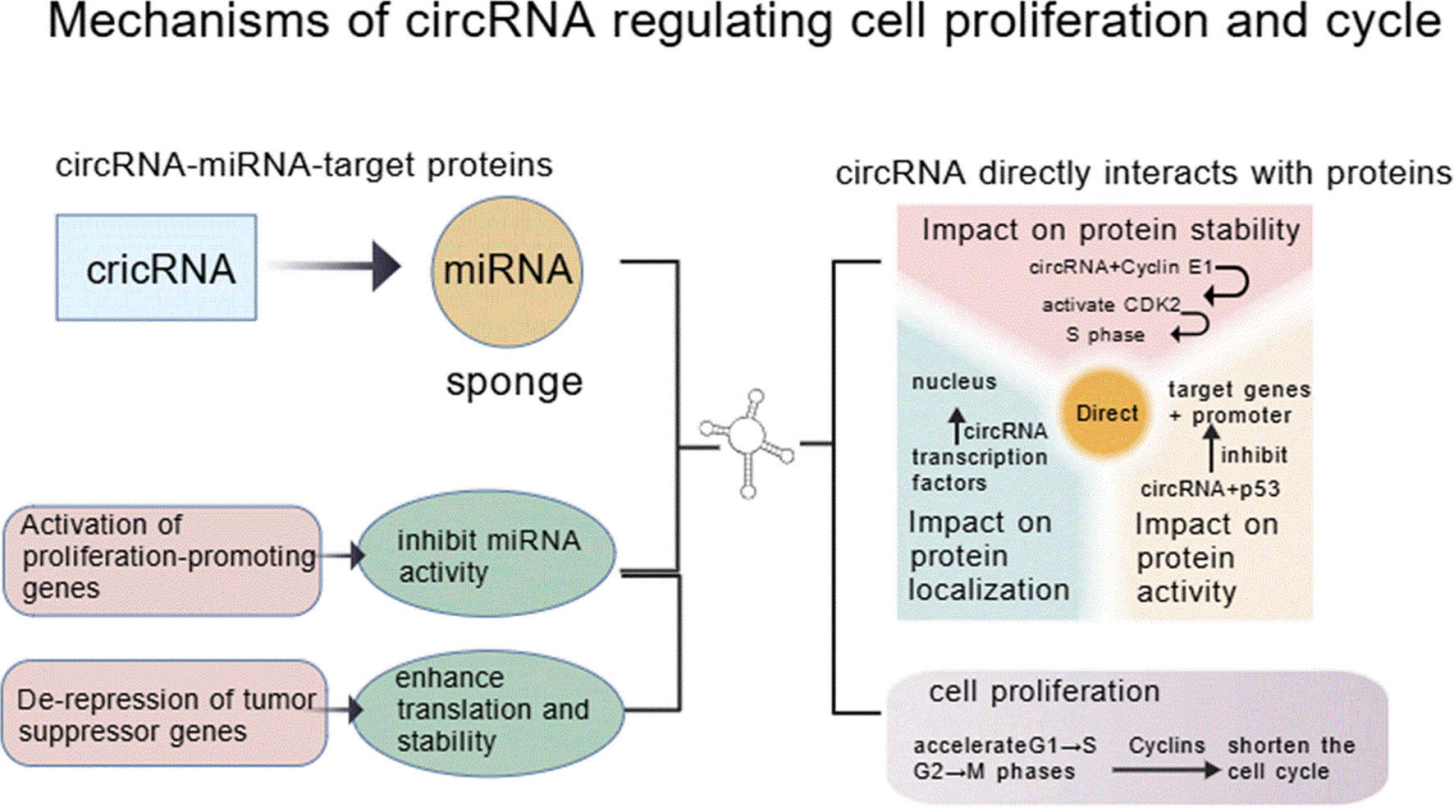
Mechanisms by which circRNAs regulate cell proliferation and cell cycle in HNSCC.

In their 2020 study, Wang et al. first observed high expression abundance of circRNA_103862 in LSCC tissues, which was associated with LSCC patients’ prognosis and metastatic status; subsequent functional experiments showed that silencing circRNA_103862 impairs LSCC cell proliferation, migration, and invasion potential, and rescue experiments validated that circRNA_103862 exerts oncogenic effects through regulating miR-493-5p—additionally, luciferase reporter assays confirmed Golgi membrane protein 1 (GOLM1) acting as a downstream target of miR-493-5p. Collectively, these findings reveal that circRNA_103862 drives LSCC progression and metastasis via acting on the miR-493-5p/GOLM1 axis, highlighting its potential to serve as a diagnostic biomarker and therapeutic target for LSCC (18). As outlined in **Figure 2**, circRNAs contribute to HNSCC progression through multiple interconnected mechanisms, including modulation of oncogenic signaling pathways, immune evasion, and therapeutic resistance.

### 3.2 Apoptosis and autophagy

Gao et al. reported that in 100 LSCC patient samples, elevated circPARD3 levels correlated with advanced T stage (p < 0.05), N stage (p = 0.001), and clinical stage (p < 0.001) as well as with poor tumor differentiation (p = 0.025) and poor patient prognosis (p = 0.002); in terms of function, circPARD3 suppresses autophagy and enhances the proliferation, migration, invasion, and chemoresistance of LSCC cells, and additional investigations suggested that the core mechanism underlying these roles of circPARD3—inhibiting autophagy, promoting LSCC progression, and triggering chemoresistance—involves the sponging of miR-145-5p, thereby triggering the activation of the PRKCI-Akt-mTOR signaling pathway (19).

### 3.3 Invasion-metastasis and EMT

Chen et al. (2022) reported that circSHKBP1 exhibited elevated expression in both LSCC clinical specimens and LSCC cell lines. Overexpression of circSHkbp1 was strongly associated with poor prognosis. The overexpression of circshkbp1 was positively correlated with Cellular processes including proliferation, invasion, angiogenesis, the development of a stem-like phenotype, and the progression of tumor growth. MIR-766-5p was downregulated in LSCC samples and negatively correlated with circshkbp1. HMGA2 was upregulated in LSCC samples and positively correlated with circshkbp1Levels of circSHKBP1, miR-766-5p, and HMGA2 were associated with clinical characteristics of tumor patients, including lymph node metastasis status and TNM staging. Mechanistic investigations demonstrated that circSHKBP1 exerts a binding interaction with miR-766-5p, which in turn leads to the inhibition of HMGA2—the target gene of miR-766-5p; additionally, in LSCC models, the suppressive impact on cancer cells induced by circSHKBP1 knockdown was rescued by two interventions: the inhibition of miR-766-5p and the overexpression of HMGA2 (20). In summary, circSHKBP1 promotes the occurrence of LSCC by targeting miR-766-5p.

Gong et al. (2022) observed that circBFAR expression was elevated in both LSCC tissues and cell lines, and this upregulated expression was associated with the progression of clinical stages and overall survival outcomes in LSCC patients. Silencing circBFAR (via knockdown) exerted inhibitory effects on LSCC cell viability and proliferation, while also blocking the migratory and invasive capabilities of these cells. Additionally, Silencing of circBFAR suppressed tube formation in LSCC cells and decreased the protein expression levels of Ki-67, matrix metalloproteinase 2 (MMP2), and vascular endothelial growth factor A (VEGFA). Subsequent experiments identified miR-31-5p as a target molecule of circBFAR; notably, downregulating miR-31-5p reversed the inhibitory impacts induced by circBFAR deficiency—including the suppression of LSCC cell viability, proliferation, migration, invasion, and tube formation, as well as the reduced protein expression of Ki-67, and VEGFA. Further investigations revealed that collagen type V alpha 1 chain (COL5A1) is negatively regulated by miR-31-5p and exhibits upregulated expression in LSCC tissues and cells. Moreover, overexpressing COL5A1 was found to counteract the inhibitory effect of miR-31-5p on LSCC cells (21).The study concluded that circBFAR deficiency suppressed *in vivo* tumor growth.

### 3.4 Cancer stem cell stemness

Rong L et al. (2022) first demonstrated that upregulated circZDBF2 facilitates the malignant behaviors of OSCC cells, including cell proliferation, invasion, migration, and EMT. Furthermore, the study suggested that in OSCC cells, circZDBF2 acts as a competing endogenous RNA (ceRNA); it exerts this role by sponging miR-362-5p and miR-500b-5p, a process that ultimately leads to the upregulation of RNF145 expression levels. Moreover, circZDBF2 recruits the transcription factor CEBPB to upregulate RNF145 expression. The study also confirmed that RNF145 activates the NFκB signaling pathway and regulates IL-8 transcription in oral squamous cell carcinoma (OSCC) (22).

### 3.5 Immune evasion

In 2024, Ge and his colleagues identified a significant inverse correlation between circE7—a circular RNA encoded by human papillomavirus (HPV)—and the infiltration of CD8+ T cells in head and neck squamous cell carcinoma (HNSCC) tumors. Through *in vitro* and *in vivo* experimentation, the study demonstrated that circE7 transcriptionally represses LGALS9—the gene encoding galectin-9—thereby impairing T cell functionality and viability. Mechanistically, This process entails circE7 binding to acetyl-CoA carboxylase 1 (ACC1), an interaction that induces the dephosphorylation and activation of ACC1. Once activated, ACC1 in turn diminishes H3K27 acetylation at the LGALS9 promoter region, ultimately resulting in reduced galectin-9 expression. Notably, galectin-9 interacts with the immune checkpoint receptors TIM-3 and PD-1, in turn inhibiting the secretion of cytotoxic cytokines by T cells and facilitating T cell apoptosis. By delineating how HPV utilizes circE7 to induce epigenetic reprogramming that facilitates immune evasion in HNSCC, this work proposes a novel therapeutic strategy: combining anti-PD-1 and anti-TIM-3 inhibitors to counteract this immunosuppressive axis. This approach targets both the galectin-9-mediated immune checkpoint blockade and the underlying epigenetic dysregulation driven by HPV, offering a potential avenue to enhance immunotherapy efficacy in HNSCC (23). While the study by Ge et al. (2024) elucidates a novel immune evasion mechanism, its findings are based on specific HPV-positive models. Whether this mechanism is ubiquitous in HPV-negative HNSCC, and how it interacts with other immune checkpoints, remains an open question and a crucial area for future investigation.

### 3.6 Metabolic rewiring

In their 2022 research, Long and colleagues examined the expression profiles of circ_0008068, Katanin p60 ATPase subunit A-like 1 (KATNAL1) mRNA, miR-153-3p, and Acylglycerol kinase (AGK) via quantitative real-time polymerase chain reaction (qRT-PCR) and Western blotting. They also conducted systematic *in vitro* and *in vivo* assays to thoroughly investigate how circ_0008068 influences the biological behaviors of OSCC. For the assessment of interactions between miR-153-3p and circ_0008068, as well as between miR-153-3p and AGK, dual-luciferase reporter gene experiments and RNA immunoprecipitation (RIP) assays were applied. The study found that in OSCC tissues or cells, circ_0008068 and AGK exhibited increased expression, whereas miR-153-3p showed decreased expression. Moreover, the knockdown of circ_0008068 led to inhibited proliferation, migration, invasion, and tube formation of OSCC cells, and promoted the apoptotic process of OSCC cells (24). It was verified in the study that the binding of circ_0008068 to miR-153-3p mediates the regulation of AGK expression—with AGK being the target of miR-153-3p—and this regulatory cascade ultimately drives an increase in glycolytic activity.

### 3.7 Chemoresistance

Beyond the well-established roles in proliferation and metastasis, circRNAs are increasingly recognized as pivotal regulators of chemoresistance in HNSCC, presenting a significant barrier to successful treatment. They orchestrate resistance through a multifaceted network of mechanisms, primarily functioning as miRNA sponges to de-repress pro-survival and anti-apoptotic target genes. For instance, specific circRNAs (circRNA_100284) have been shown to sequester miRNAs like miR-10a, subsequently activating pathways involved in autophagy and DNA damage repair that confer resistance to agents such as cisplatin (25). Additionally, their interaction with RBPs and potential to encode functional peptides further expand their arsenal in promoting drug efflux and enhancing cell survival under therapeutic stress. The horizontal transfer of exosomal circRNAs between tumor cells also disseminates resistant phenotypes, highlighting a novel mechanism of community-wide adaptation. Targeting these therapy-induced circRNAs could therefore be a promising strategy to re-sensitize tumors and improve clinical outcomes.

In summary, the multifaceted roles of circRNAs in driving HNSCC malignancy are complex and extensive. To provide a clearer overview, we have consolidated the key circRNAs, their mechanisms of action, functional consequences, and clinical relevance in **Table 1**.

**Table 1:** Key circular RNAs in HNSCC: mechanisms, functions, and clinical implications.

circRNA Name	Regulation	Expression in HNSCC	Molecular mechanism	Functional role in HNSCC	Clinical significance	Key references
circPVT1	↑	↑ in tumor tissues (esp. with TP53 mut)	Sponges miR-497-5p Interacts with mut-p53/YAP/TEAD complex	Promotes cell proliferation	Prognostic: Poor OS. Therapeutic: siRNA/shRNA silencing inhibits tumor progression.	Verduci L, et al., 2017 (17)
circRNA_103862	↑	↑ in LSCC tissues	Sponges miR-493-5p to upregulate GOLM1	Drives proliferation, migration, invasion	Diagnostic/Prognostic: Associated with metastasis and poor prognosis in LSCC.	Wang X, et al., 2020 (18)
circPARD3	↑	↑ in LSCC tissues	Sponges miR-145-5p to activate PRKCI-Akt-mTOR pathway	Suppresses autophagy, promotes proliferation, migration, invasion, chemoresistance	Prognostic: Correlates with advanced T/N stage, poor differentiation, and poor OS. Therapeutic: Potential target to overcome chemoresistance.	Gao W, et al., 2020 (19)
circSHKBP1	↑	↑ in LSCC tissues and cell lines	Sponges miR-766-5p to upregulate HMGA2	Promotes proliferation, invasion, angiogenesis, stemness	Prognostic: Associated with lymph node metastasis, TNM stage, and poor prognosis.	Chen F, et al., 2022 (20)
circBFAR	↑	↑ in LSCC tissues and cell lines	Sponges miR-31-5p to upregulate COL5A1	Promotes cell viability, proliferation, migration, invasion, tube formation	Prognostic: Associated with advanced clinical stage and poor OS.	Gong H, et al., 2022 (21)
circZDBF2	↑	↑ in OSCC cells	• Sponges miR-362-5p/miR-500b-5p • Recruits CEBPB to upregulate RNF145	Promotes proliferation, invasion, migration, EMT; Activates NF-κB pathway	Functional: Key regulator of cancer stem cell stemness and tumor progression.	Rong L, et al., 2022 (22)
circE7 (HPV-encoded)	↑	↑ in HPV+ HNSCC	Binds to ACC1, inducing dephosphorylation → reduces H3K27ac at LGALS9 promoter → suppresses Galectin-9	Facilitates immune evasion by impairing CD8+ T cell function and viability	Therapeutic: Novel target for immunotherapy. Suggests combo therapy with anti-PD-1 & anti-TIM-3 inhibitors.	Ge J, et al., 2024 (23)
circ_0008068	↑	↑ in OSCC tissues/cells	Sponges miR-153-3p to upregulate AGK	Promotes proliferation, migration, invasion, tube formation, glycolytic activity	Functional: Drives metabolic rewiring (glycolysis) in OSCC.	Long Y, et al., 2022 (24)
hsa_circ_0036722	↑	↑ in LSCC tissues	–	–	High diagnostic efficacy (AUC = 0.838) for distinguishing LSCC from normal tissues.	Guo Y, et al., 2020 (26)
circ_0000199	↓	↓ in OSCC plasma exosomes	Sponges miR-135a-5p (inferred from function)	Inhibits proliferation, promotes apoptosis (*in vitro*)	Diagnostic: Salivary exosomal biomarker for OSCC (AUC = 0.825). Prognostic: Low levels correlate with poor survival.	Luo Y, et al., (27)
hsa_circ_100855	↑	↑ in LSCC tissues	–	–	Prognostic: Associated with lymph node metastasis and advanced clinical stage.	Kan X, et al., 2016 (28)
hsa_circ_104491	↓	↓ in LSCC tissues	–	–	Prognostic: Low expression linked to advanced T stage, lymph node metastasis, advanced clinical stage, and poor differentiation.	Kan X, et al., 2016 (28)

↑, Upregulated; ↓, Downregulated; OS, Overall Survival; LSCC, Laryngeal Squamous Cell Carcinoma; OSCC, Oral Squamous Cell Carcinoma; -, Not specified in text.

## 4 circRNA as diagnostic and prognostic biomarkers for HNSCC

### 4.1 Differential expression profiles at the tissue level

Wang et al. (2018) conducted high-throughput microarray analysis to detect circRNA and mRNA expression in HNSCC tissues. A total of 12,366 circular RNAs (circRNAs) and 35,252 messenger RNAs (mRNAs) were detected in five pairs of HNSCC and normal tissues. Among these identified molecules, 287 were classified as differentially expressed circular RNAs (circRNAs); specifically, 146 of these circRNAs showed upregulated expression, while the remaining 141 exhibited downregulated expression (13).

Wu et al. conducted gene ontology (GO) enrichment analysis specifically for each individual cell cluster in their study. They found that processes involving the proliferation of epithelial cells were more prevalent in clusters with lower epithelial-mesenchymal transition (EMT) scores. On the other hand, cell migration and mesenchymal-related biological events were more prominent in those cell clusters characterized by higher EMT levels (14). This confirmed at the single-cell level that circRNA expression is significantly heterogeneous among tumor-infiltrating immune cells.

### 4.2 Diagnostic efficacy

Guo et al. stated that when using hsa_circ_0036722 to distinguish LSCC from adjacent normal tissues, the area under the ROC curve (AUC) was 0.838 (95% CI: 0.750–0.925). With an optimal threshold of 0.5, sensitivity was found to be 82.9%, while specificity was found to be 75.0% (26). These pilot data suggest that hsa_circ_0036722 warrants further evaluation as a candidate diagnostic biomarker for LSCC in larger, multicenter cohorts. Luo et al. (2020) evaluated the diagnostic ability of salivary exosomal circ_0000199 for OSCC using ROC curve analysis. The results showed an area under the curve (AUC) of 0.825 (95% confidence interval (CI): 0.752–0.898), with a sensitivity of 80.0% and a specificity of 78.6% (27). This suggests that salivary exosomal circ_0000199 could be a potential diagnostic biomarker for OSCC.

### 4.3 Prognostic value

Nath et al. included 9 high-quality studies in a meta-analysis. The results showed that a meta-analysis of nine studies (1,368 patients) indicated an association between elevated expression of seven circRNAs and shorter overall survival (pooled HR = 3.40, 95% CI 2.66–4.36); however, heterogeneity and publication bias require further assessment. The reduced expression of two circular RNAs, namely circ0092125 and circ00072387, was correlated with an unfavorable prognosis in patients diagnosed with HNSCC, with a hazard ratio (HR) of 2.83 and a 95% confidence interval (CI) ranging from 1.73 to 4.65 (29).

### 4.4 Association with clinicopathological parameters

Gao et al. reported that high circPARD3 levels were positively associated with two key clinicopathological features: the T stage of the disease and cervical lymph node metastasis (referred to as N stage). The expression level of circPARD3 gradually increased from T1 to T4 stages. Furthermore, circPARD3 levels were higher in poorly differentiated LSCC tissues than in well-differentiated tissues. The data validated that increased circPARD3 expression showed a positive correlation with three key clinical indicators: tumor T stage, lymph node metastasis status, and shorter OS, where the HR was 3.93 (19).

### 4.5 Non-invasive detection in body fluids

With features like widespread occurrence, evolutionary conservation, and stable expression levels in saliva, blood, and exosomes, circRNAs hold significant promise as biomarkers for the early identification and prognostic evaluation of tumors (25). One illustrative example is circMAN1A2: it exhibits high expression in NPC cell lines, and its serum levels are markedly higher in NPC patients compared to healthy individuals—hinting at its potential role in the early detection of nasopharyngeal carcinoma (30). Likewise, the expression of exosomal circMYC enables the differentiation of radiation sensitivity in nasopharyngeal carcinoma (NPC) patients (31), whereas the upregulation of circSERPINA3 is linked to NPC progression—including lymph node metastases and worse survival outcomes (32).

Kan et al. employed microarray analysis to investigate the comprehensive circular RNA (circRNA) expression profile in LSCC tissues, with adjacent non-tumor tissues serving as the control group for comparison. They found that 698 circRNAs were present at different levels in LSCC tissues (28). Subsequent qRT-PCR analysis showed that has-circRNA-1008555 was the most upregulated, while has-circRNA-1044912 was downregulated in LSCC. The levels of has-circRNA-1008555 were significantly higher in laryngeal squamous cell carcinoma (LSCC) patients with metastasis to the cervical lymph nodes or at an advanced clinical stage. On the other hand, has-circRNA-1044912 exhibited notably reduced expression in LSCC patients with T3–4 stage tumors, cervical lymph node metastasis, advanced clinical stages, or tumors with poor differentiation (28). These findings point to an association between circRNAs and LSCC staging as well as prognosis. In conclusion, the discovery of circRNAs holds significant promise for disease diagnosis and outcome prediction in HNSCC.

## 5 Therapeutic translation of circRNA

### 5.1 Targeted therapy

Preliminary *in-vitro* and mouse studies suggest that selected circRNAs might be exploitable as therapeutic targets; efficacy, delivery specificity and safety profiles remain to be established in clinically relevant models. For instance, circ-Ccnb1 has been identified to inhibit cell invasion and tumorigenesis through the dissociation of the Ccnb1/Cdk1 complex. Furthermore, circ-ZNF532 plays a key role in regulating processes such as pericyte degradation and vascular dysfunction associated with diabetes (33).

### 5.2 Gene silencing and RNA interference

Silencing specific circRNA using siRNA or shRNA technology can inhibit tumor progression. For instance, silencing circPVT1 has been demonstrated to markedly suppress tumour progression (34). Moreover, CRISPR/Cas9 has been used to target and eliminate circHIPK3 specifically *in vitro* without affecting its linear mRNA, thus inhibiting cell proliferation (33).

### 5.3 Nanoliposome delivery system

Nanoliposomes can serve as a delivery system for circRNA therapy, enhancing the stability and targeting of drugs. Du et al. devised a treatment for breast cancer involving the delivery of gold nanorods conjugated with siRNA that target circDnmt1. These nanorods are able to interact with the Auf1 and p53 proteins (35).

### 5.4 Exosome-mediated therapy

Exosomes are currently being investigated for use as both targeting agents and carriers of circRNA expression (36). Exosomes naturally contain a diverse array of molecules, such as circRNA, miRNA, long non-coding RNA, proteins, lipids, and DNA fragments. It has been shown that exosomal circRNA facilitates cancer development by promoting cell proliferation, tumour metastasis and drug resistance (37). Exosomes can transport circRNA for non-invasive diagnosis and therapeutic applications. For instance, in patients with OSCC, circulating exosomal circ_0000199 levels are significantly elevated and correlate with TNM staging. The overexpression of circ_0000199 has been shown to encourage cell growth and hinder cell death, while the inhibition of circ_0000199 has the reverse effect. These findings suggest that circulating exosomal circ_0000199 possesses utility as a diagnostic marker and constitutes a feasible therapeutic target for oral squamous cell carcinoma (OSCC) (27).

### 5.5 Translation regulation and protein expression

**Figure 3:**
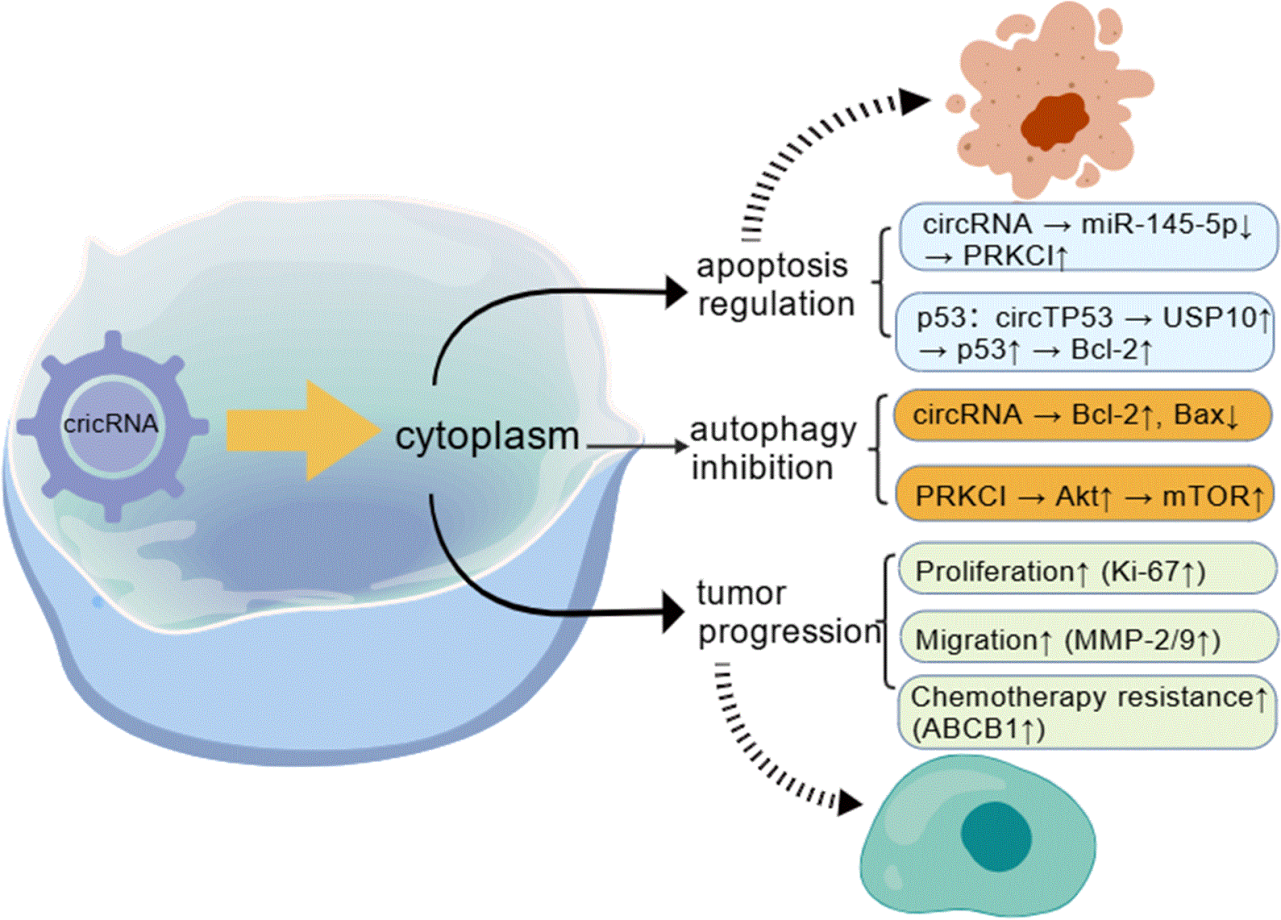
Clinical translation potential of circRNAs in HNSCC.

The translational regulatory functions of circRNA have also been investigated for therapeutic applications. Specific circular RNAs (circRNAs)—including circZNF609, circMb1, circ-FBXW7, circPINTexon2, and circ-SHPRH—are capable of exerting regulatory functions by triggering translation in the untranslated region (UTR). This translational initiation is facilitated either through an internal ribosome entry site (IRES) or N6-methyladenosine (m^6^A) modification (4). **Figure 3** illustrates the potential clinical translation of circRNAs in HNSCC.

## 6 Challenges and prospects

Current research on circRNA primarily focuses on its role as a miRNA sponge, while its effects on key biological processes in HNSCC—including transcription, splicing, protein-protein interactions, and the ability to encode proteins or peptides—have not been sufficiently investigated. For instance, circE7 inhibits the phosphorylation and activation of ACC1, thereby reducing intracellular acetyl-CoA levels and epigenetically suppressing the expression of the LGALS9 gene, leading to tumor immune evasion. Given the complexity of these actions, further investigation is necessary to confirm these mechanisms (23). The upstream regulatory mechanisms underlying circRNA expression dysregulation, such as alternative splicing and post-transcriptional modifications, still require exploration. For instance, circPVT1 promotes HNSCC by interacting with other tumors and binding differently to the p53/YAP/TEAD transcribing complex. However, the underlying regulatory processes are still unclear (17). Moreover, most studies on circRNA biomarkers are based on small sample sizes. While these preliminary findings are promising, their value in early diagnosis and prognostic evaluation needs to be confirmed through validation with larger, multicenter clinical samples. CircRNA may hold potential for non-invasive diagnosis in HNSCC, but this hypothesis requires rigorous testing.

circRNA may also emerge as a new target for immunotherapy. CircF7 facilitates immune system evasion in HNSCC by inhibiting the immune system checkpoint protein Galectin9. This suggests that the combination of TIM-3 and PD-1 antibody immunotherapy could potentially enhance the effectiveness of HNSCC treatment. However, these findings are still in the early stages and need to be validated through further experimental and clinical studies (23). Future studies should combine multi-omics data, such as transcriptomics, proteomics and metabolomics, to thoroughly elucidate the role that circRNAs play in HNSCC. Developing efficient circRNA delivery vehicles, such as nanoliposomes and exosomes, will advance the clinical translation of circRNA. Establishing multicenter, international HNSCC-circRNA databases will help standardize circRNA detection and facilitate its clinical translation. However, these efforts are still in the exploratory phase and face significant challenges. Future research should leverage the growing power of artificial intelligence and machine learning in circRNA research. Integrating multi-omics data (transcriptomics, proteomics) with predictive computational models, such as the DeepDCR algorithm (38), will be essential for constructing comprehensive circRNA-regulated networks in HNSCC, systematically elucidating their roles in tumorigenesis, and identifying the most therapeutically actionable targets.

In summary, research on circRNA in HNSCC faces significant challenges, particularly in elucidating complex biological mechanisms and achieving clinical translation. While technological advancements and multidisciplinary integration offer promise, many of the proposed mechanisms and applications of circRNA in HNSCC remain hypothetical and require extensive experimental validation. The current review highlights these potential roles and underscores the need for further research to overcome the limitations of existing studies and to fully realize the diagnostic and therapeutic potential of circRNA in HNSCC.
